# Polymerisation Shrinkage Profiling of Dental Composites using Optical Fibre Sensing and their Correlation with Degree of Conversion and Curing Rate

**DOI:** 10.1038/s41598-019-40162-z

**Published:** 2019-02-28

**Authors:** Ginu Rajan, Raju Raju, Sagar Jinachandran, Paul Farrar, Jiangtao Xi, B. Gangadhara Prusty

**Affiliations:** 1 School of Electrical, Computer and Telecommunications Engineering, University of Wollongong, Wollongong, Australia; 2 School of Mechanical and Manufacturing Engineering, UNSW Sydney, Sydney, Australia; 3 SDI Ltd, Bayswater, Victoria, Australia

## Abstract

Traditional polymerisation shrinkage (PS) measurement systems measure average PS of dental composites, but the true local PS varies along the length and breadth of the composite. The PS depends on the curing light intensity distribution, resultant degree of conversion (DOC) and the curing rate. In this paper, optical fibre Bragg grating (FBG) sensing based technology is used to measure the linear post-gel PS at multiple locations within dental composite specimens, and is correlated with DOC and curing rate. A commercial dental composite is used, and its post-gel PS and DOC are mapped using embedded fibre Bragg grating sensors at different curing conditions. The distance between the curing lamp and the composite specimen is varied which resulted in different intensity distribution across the specimen. The effect of curing light intensity distribution on PS, curing rate and DOC are investigated for demonstrating a relationship among them. It is demonstrated that FBG sensing method is an effective method to accurately profiling post-gel PS across the specimen.

## Introduction

Focus on aesthetics, toxicity concerns and ease of application has led methacrylate based dental composites to be the preferred restorative material by practitioners around the world^1^. Current drift towards ‘minimal invasive dentistry’ has increased the usage of dental composites at all locations of mouth area. Applications of adhesive restorative composites in dentistry are multifold, ranging from root canal posts, posterior restoration, tooth prostheses and orthodontic devices to cavity liners, inlays, crowns and onlays^1^. However, the main drawback is their contraction during/after polymerisation^2^.

Methacrylate based dental composites are generally photocured under blue-light at a wavelength range of 420–480 nm. During photo-curing, double carbon links (C=C) in monomer are converted to single links (C-C) in polymer; the number of molecules converted to polymer is referred as degree of conversion (DOC)^3^. Mechanical and physical performance of dental resin composites are directly dependent on the extent of DOC during polymerisation; higher the conversion, higher are the longevity, mechanical and physical properties. Nonetheless, most of the dental resins show considerable amount of monomers remaining in the cured polymers^4–6^, which could be due to the influence of several factors such as irradiation time, irradiation distance, type of light source, size of light tip, optimum wavelength, power density, type of monomers, size and volume fraction of fillers, refraction coefficients of both organic matrix and inorganic fillers, type and quantity of photo-initiators and co-initiators^7,8^.

Polymerisation process involves certain amount of polymerisation shrinkage (PS), which could be due to chemical, thermal or post contraction^9^. During chemical contraction, the van der Waals distance between the atoms of monomers are reduced from to ~10^4^ Å ~1.0 Å, resulting in bulk contraction in cured resin, i.e., volumetric polymerisation shrinkage^10–12^. Though the effect of thermal contraction due to exothermic reaction and cooling back to room temperature is minimal, internal stresses could be induced which could be detrimental to the restoration^9^. During the initial 24 hrs after photo-polymerisation, conversion degree is not significantly affected, but the concentration of free radicals is largely reduced, leading to post-cure shrinkage^13^. Typical volumetric shrinkage of dental composites is generally in the range of 1–6%^14–16^.

Considerable literature exists on the DOC, PS and their effect on physical and mechanical properties of dental composites^7,17–22^. An ideal dental composite is expected to have a higher DOC and minimum PS. A direct correlation exists between DOC and PS, higher the conversion of C=C bonds to C-C bonds, lower is the volumetric contraction during polymerisation^9^.

Various methods exist to measure volumetric PS of dental resins such as mercury dilatometer^23^ or water filled dilatometer^24^, density based gas pycnometer^25^, buoyancy (density in water) based measurements^26^, Archimedes approach with buoyant force principle^27^, electro-magnetic balance^28^, video imaging technique^29^ and computed tomography (CT) based technique^30^.

Assuming the dental resin is macroscopically isotropic, shrinkage is also assumed to be equal in all directions, i.e., volumetric shrinkage is assumed to be thrice of linear shrinkage^31^. Linear shrinkage of dental resins could be measured using dilatometer^32^, bonded disc based Watts method^32^, strain gauges^33^, scanning laser beam method^34^, video imaging technique^35^, linear displacement and force^36^ and by using fibre optic sensors^12,37,38^.

Initial curing rate of photo-cured dental composite also has significant influence in PS. Higher shrinkage leads to higher shrinkage stresses and lower bonding efficiency, reducing the mechanical and physical properties of the restorative material leading to microleakage and failure of the restoration. Also the total shrinkage in a dental composite could be divided into pre-gel (viscous to gel) and post-gel (gel to solidification) phases^39,40^. During pre-gel phase the material still possess fluidity and flowability i.e., any shrinkage/contraction would result in deformation of the material and with negligible stress. However, when the crosslinking reaches its apex in post-gel phase, the gel is converted to solid (higher elastic properties) and any contraction would result in minimal deformation and maximum shrinkage stress^41,42^. Among the various conventional PS measurement methods strain gauge neglects any deformation of viscous flow in pre-gel phase and measures the clinically significant post-gel deformation. Multiple studies have confirmed the usage of strain-gauges for efficient measurement of volumetric shrinkage^39,41–44^.

In this study, fibre optic sensor based approach is used to measure the linear post-gel PS of dental composite resins. Fibre-optic sensing technology is a powerful and potentially rich technology that is currently implemented in a wide variety of applications, where the optical fibre itself is acting as the sensor head. This technology can provide novel solutions to many challenging instrumentation requirements of the medical/dental industry and is recognised as one of the most promising approaches to sensing a wide variety of measurands. Fibre optic sensors have greater advantages over conventional measurement techniques due to their low weight, small size, immunity to electromagnetic interference, resistant to high temperature and chemically reactive environment, high sensitivity and multiplexing and able to measure various parameters such as displacement, temperature, strain, force, acoustic emission, etc.^45^. Among the various types of fibre optic sensors available, fibre Bragg grating (FBG) is preferred sensor for composites^46^ due to ease of embedding the sensor within the composite. Polymerisation shrinkage, water sorption, thermal expansion and setting expansion, are successfully measured in the past^12,37,38,47^. In this paper, PS profile mapping of the composite using an FBG array, correlation between DOC, rate of cure and linear PS in real-time during photo-polymerisation process of a commercial dental composite using optical fibre sensing method is investigated

## Materials and Methods

### Dental Composite Specimens

A commercial dental composite resin Shofu Beautifil FO3 is used as the test specimen in this study. Beautifil F03 is a flowable medium viscosity giomer nano-hybrid flowable composite (67.3% filler loading) with a polymerisation shrinkage value of 3.4%^48^ and flexural modulus of 7.6 GPa^49^.

### Curing Lamp Intensity Profile Measurement

**Figure 1:**
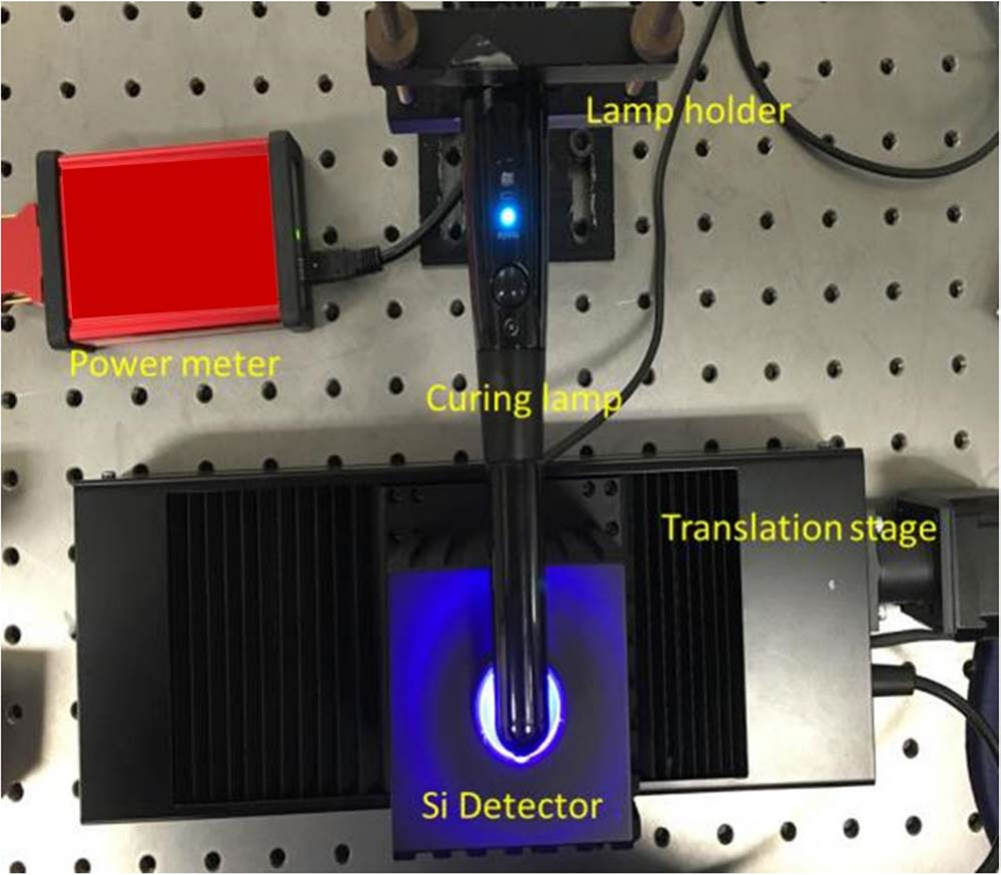
Experimental setup to measure the intensity profile of the curing lamp.

The curing lamp used in this study is X-Lite-II, which has a broad emission wavelength of 385–515 nm with a manufacturer specified intensity of 1700 mW/cm^2^. The lamp head diameter was 8 mm and was placed on a linear translation stage. To measure the intensity profile of the lamp, a power meter based setup is used as shown in Fig. 1. The sensor used was S142C (Thorlabs) which has a measurement range of (300–700 nm) connected to a power energy meter PM100USB (Thorlabs). To measure the lamp profile, a pin-holed mount was placed on the power meter sensor (where pin hole is in the middle of the sensor) and was scanned edge to edge of the lamp head.

### Fibre Bragg Grating Sensors

An elementary FBG is a short section of single-mode optical fibre in which the core refractive index is modulated periodically using an intense optical interference pattern, typically at UV wavelengths. This periodic index modulation enables the light to be coupled from the forward propagating core mode into backward propagating core mode generating a reflection response having a central wavelength *λ*_*G*_ (Bragg wavelength), is given by^50^,

1 $${\lambda }_{G}=2{n}_{eff}{\rm{\Lambda }},$$

where *n*_*eff*_ is the effective refractive index of the core and Λ is the periodicity of the refractive index modulation.

The basic principle of operation of an FBG based sensor system is to monitor the strain and temperature induced Bragg wavelength shift. The strain sensitivity of the Bragg wavelength arises from the change in period of the fibre coupled with a change in refractive index arising from the strain-optic effect, while the sensitivity temperature arises from the change in period associated with the thermal expansion of the fibre, coupled with a change in the refractive index arising from the thermo-optic effect.

The wavelength shift, Δ*λ*_*S*_, for the measurement of applied uniform longitudinal strain, Δ*ε*, is given as,

2 $$\,{\rm{\Delta }}{\lambda }_{s}={\lambda }_{G}(1-{\rho }_{e}){\rm{\Delta }}\varepsilon ,$$

where *ρ*_*e*_ is the photo elastic coefficient. For a silica core fiber the value of (1 − *ρ*_*e*_)is usually 0.78.

Thus by measuring the shift in the peak reflected wavelength of the FBG, linear PS strain and the corresponding PS value (%) of the dental composite can be obtained.

**Figure 2:**
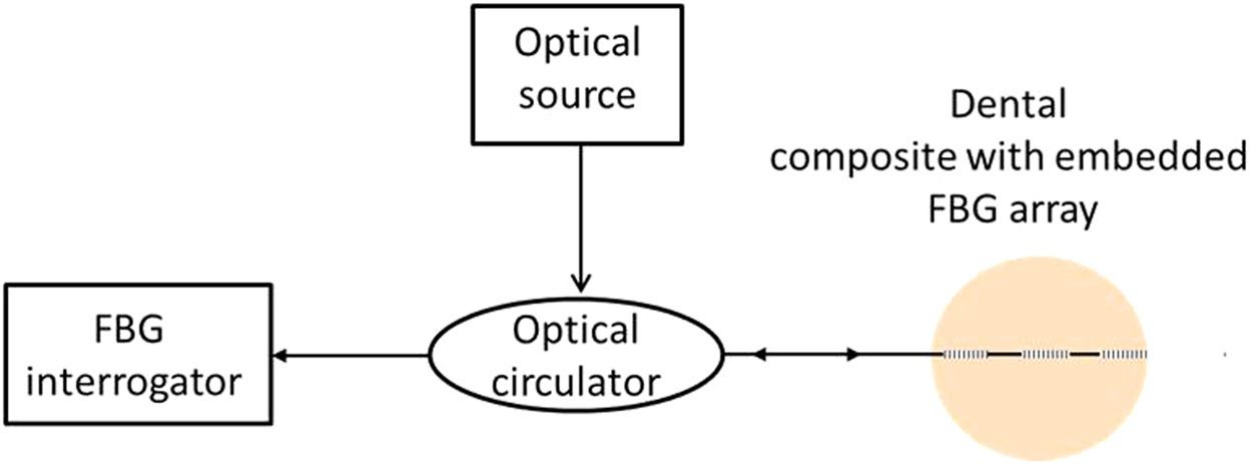
Schematic diagram of the FBG sensor based characterization system.

The FBG sensor used in this study is an array of three FBGs with a length of 3 mm with a spatial separation of 2 mm between the gratings. The free space peak reflected wavelength of the FBGs were circa 1540 nm, 1550 nm and 1560 nm respectively with a reflectivity greater than 70%. The gratings were fabricated on single mode silica fibre with polyimide buffer coating and has a diameter of circa 150 microns (supplied by DK Photonics Ltd, China). A commercial FBG interrogator IMON 256 from Ibsen Photonics A/S, which has a capability to resolve 5 pm wavelength change with a data acquisition rate of 6 kHz is used to acquire the data from the FBG sensors. A broadband source with a spectral range of 1530–1570 nm was used and the reflected signal from the FBG was directed to the interrogator via fibre optic circulator. A schematic diagram of an FBG interrogation used in this study is depicted in Fig. 2.

### Polymerisation Shrinkage Profiling and Curing Rate Measurement

**Figure 3:**
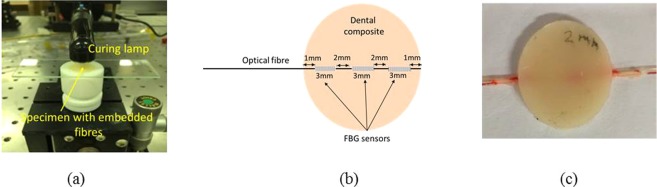
(**a**) Experimental setup for curing (**b**) schematic of the location of the FBG sensors within the specimen (**c**) photograph of a cured specimen with embedded FBG array.

A custom made Teflon mould of 15 mm and 2 mm depth is used for specimen manufacture. To embed the FBG sensor into the specimen, a 1 mm cut was made on the teflon mould surface. Once the dental composite is filled in the mould cavity, a glass slide is pressed against the mould to squeeze out excessive material and then photocured using the curing light as shown in Fig. 3(a). A schematic of the location of the FBG sensor within the specimen is shown in Fig. 3(b). Three curing conditions are tested, where the lamp is placed at 2 mm, 5 mm and 10 mm away from the specimen.

As the composite is polymerised, contraction strain induced wavelength change of the FBG sensors was recorded for 20 minutes using the setup shown in schematic Fig. 2. The reflection spectrum of the embedded FBG was also obtained before curing and after curing. The cured sample was then removed from the mould and kept safe for further studies. A photograph of the cured sample is shown in Fig. 3(c).

In order to measure the polymerisation shrinkage, initially the strain induced on the FBG sensor during the polymerisation was measured. As strain is a measure of percentage of elongation, percentage of shrinkage can be estimated from the measured strain. For this purpose all the FBGs used in the work were 3 mm long and the positioning of the FBG sensors within the material were kept to identical as much as possible and adequate pre-strain was applied to all the sensors.

### Degree of Conversion Measurement

Fourier transform infrared spectroscopy (FTIR) was used to measure the DOC of the cured specimens. Three specimens of Beautifil FO3 were prepared under three curing conditions, 2 mm, 5 mm and 10 mm distance between the lamp and the mould. Once cured, the specimens are stored at room temperature for a minimum of 24 hours, the absorbance peaks were recorded using diffuse reflection mode of the FTIR (Spotlight 400 FT-IR from PerkinElmer) over 16 scans over a wavelength range of 4000–650 cm^−1^ with a resolution of 4 cm^−1^. Absorbance of the uncured samples is also obtained under the same conditions. Three samples were prepared for each cases and FTIR data is obtained for centre and edge region for all the three set of samples (top and bottom surfaces) and is repeated for three times for each sample.

DOC was determined by estimating the changes in the peak height ratio of the absorbance intensities of aliphatic C=C peak at 1638 cm^−1^ and that of an internal standard peak of aromatic C=C at 1608 cm^−1^ during polymerisation, in relation to the uncured material. DOC at each location of the specimen was calculated using the following equation^51^;

3 $$DOC{ \% }=[1-\frac{(1638\,c{m}^{-1}/1608\,c{m}^{-1}){cured}}{(1638\,c{m}^{-1}/1608\,c{m}^{-1}){uncured}}]\ast 100,$$

## Results

### Curing Lamp Intensity and Degree of Conversion

**Figure 4:**
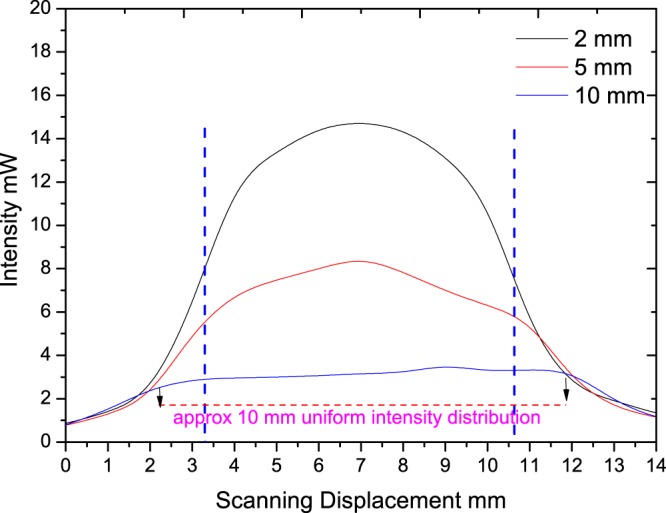
Measured intensity profile of the lamp at different distances from the detector.

Intensity profile of the lamp is measured in three scenarios, where the lamp is 2 mm, 5 mm and 10 mm away from the detector. The total intensity measured at these distances were 1300 mW/cm^2^, 961 mW/cm^2^, 667 mW/cm^2^ respectively at a set wavelength of 400 nm for the power meter. The average intensity for the whole wavelength (385–515 nm) could be different from the above. The measured curing lamp profiles under above three conditions are shown in Fig. 4. The curing lamp head diameter was 8 mm, but to measure the light intensity across specimen (diameter 15 mm), the intensity distribution across 15 mm was measured and presented.

**Figure 5:**
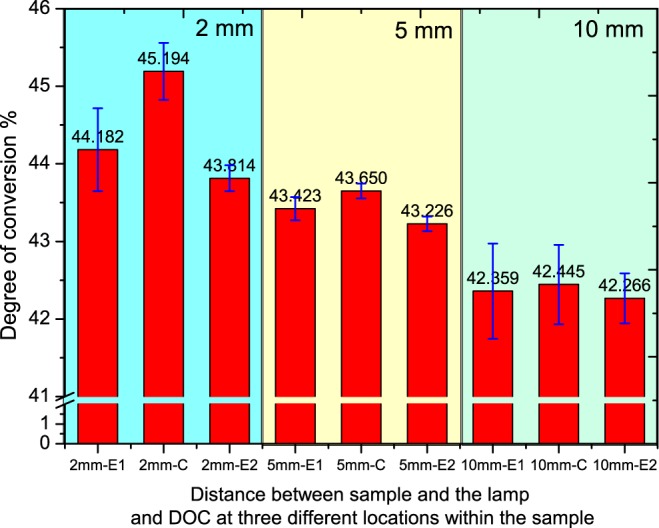
Measured DOC of the specimen at different locations (E1, E2- Edges, C- Centre) and at different distance (2 mm, 5 mm and 10 mm) from the curing lamp.

The measured average DOC from top and bottom surface of the specimens at different curing conditions and at different specimen locations are shown in Fig. 5. The corresponding standard deviations are also presented in the figure. E1 and E2 correspond to two opposite edges of specimen while C corresponds to entre of the specimen. There is a difference in the DOC at the centre and edges for case 1, which is 2 mm gap between the lamp and the mould, but is less significant for the case where the lamp is placed at 5 mm and 10 mm from the specimen. The difference in DOC of the specimens cured with lamp at 2 mm and 10 mm is approximately 2.75% for the centre region and 1.68% for the edges (average for both the edges).

### Polymerisation Shrinkage and Curing Rate

**Figure 6:**
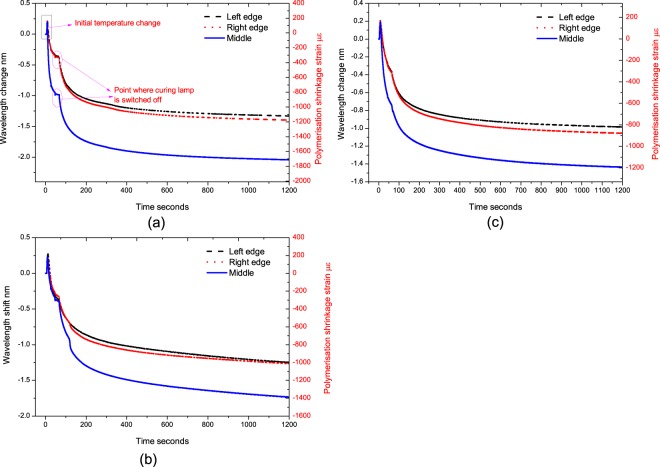
Measurement wavelength shift and polymerization shrinkage at different regions of the specimens cured at different distances from the lamp (**a**) 2 mm (**b**) 5 mm (**c**) 10 mm.

The measured curing response (wavelength change exhibited by the embedded FBG and the corresponding estimated polymerisation shrinkage strain) of the specimens at three curing conditions (2 mm, 5 mm and 10 mm from the lamp) is shown in Fig. 6(a–c). The polymerisation strain exhibited on the FBG is calculated from the change in wavelength occurred for a duration of 1200 seconds. At 60 seconds time, a small change in the response is visible due to the sudden drop in temperature when the lamp is switched off. Also the initial jump in the wavelength shift in the response is the sudden temperature change due to heat produced by the lamp as well as the exothermic reaction of the composite during photo-polymerisation, when the curing lamp is switched on. In this analysis for shrinkage estimation, the peak is considered as the initial point as this approach will eliminate the error due to temperature to a great extent. However, to improve accuracy and eliminate any discrepancies due to the temperature effect, temperature compensation methods can be applied such as, adding another FBG to the array outside the composite to monitor the temperature through the curing phase and normalizing the curing response against the temperature response will eliminate the temperature effect due to the curing lamp. However, the temperature changes due to the exothermic reaction of the composite still remains.

**Figure 7:**
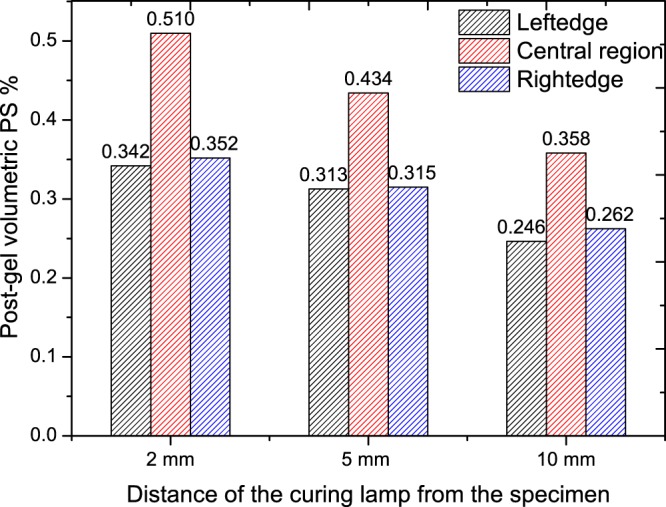
Local post-gel volumetric polymerisation shrinkage of the specimen for different curing lamp distances.

The linear post-gel PS is calculated from the polymerisation strain and post-gel volumetric PS is obtained assuming the dental composite as an isotropic material and is shown in Fig. 7. A maximum post-gel PS of 0.510% is obtained at the centre of the specimen for a curing lamp distance of 2 mm, while shrinkage of 0.358% is obtained for a curing lamp distance of 10 mm. The measured post-gel PS values at the edges are lesser compared to the centre region of the specimen. The curing rate of the specimen is also calculated by measuring the slope of the response for the first 100 seconds for all the three cases. The measured curing rate is shown in Table 1, where it is seen that the curing rate is different for different locations within the specimen as well for different distance from the curing lamp.

**Table 1:** Local curing rate of the specimen for the first 100 seconds and average for 20 minutes.

Location/Distance	Centre (%s^−1^)		Left edge (%s^−1^)		Right edge (%s^−1^)	
	100 s	20 min Av	100 s	20 min Av	100 s	20 min Av
2 mm	0.00360	0.000425	0.00214	0.000285	0.00203	0.000293
5 mm	0.00200	0.000360	0.00146	0.000260	0.00146	0.000262
10 mm	0.00240	0.000321	0.00154	0.000205	0.00162	0.000218

### Spectra of FBG sensor array before and after curing

**Figure 8:**
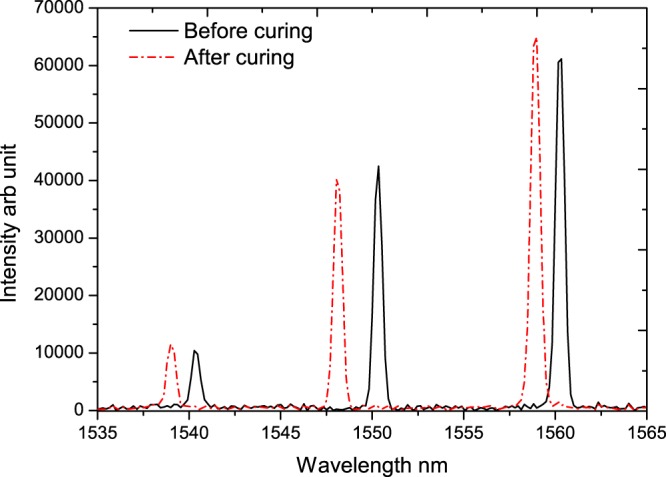
Spectral response of the FBG sensor array before and after curing.

We have also looked into the spectrum of the FBG sensor before and after curing to see any possible anomalies due to the strain gradient within the sample. The response of the FBG sensor before and after curing for the specimen cured 2 mm away from the lamp is shown in Fig. 8. As the light source is connected to the end of the FBG with 1560 nm, it shows the highest intensity and the subsequent ones show a reduced intensity. Though the PS value at the central region and edges are different there is no significant impact of strain gradient on the FBG spectrum which is due to the usage of miniature 3 mm length gratings.

## Discussions

### Impact of curing lamp intensity distribution on DOC, PS and curing rate and their correlation

It can be seen from the Fig. 4 that the curing light intensity distribution is non-uniform especially when the lamp is closer to the detector. When the lamp is 10 mm away from the detector, though the intensity is less, a uniform intensity distribution is obtained for approximately 10 mm scanning region as shown in Fig. 4. As such there is a trade-off between total intensity and intensity distribution in these cases. When the lamp is away there is a considerable decrease in the intensity received at the detector, and therefore it might affect the curing phenomena.

One of the observations from the results shown Fig. 5 is that there is no significant change for DOC at different location for specimens cured at different conditions (maximum difference is approximately 2.5%). The small difference in DOC is due to the decrease in light/power intensity with increase in distance from the restoration surface (1300 mW/cm^2^, 961 mW/cm^2^, 667 mW/cm^2^) and due to the intensity profile of the lamp. It is shown in the study by Caldas *et al*.^52^, increasing the distance between the curing tip light and restoration surface will result in lower power density. In this study, the curing time is kept constant and the light intensity is varied due to distance between light tip and restoration surface. Also previous studies on the effect of distance on irradiance and beam profile of the curing lamp and its subsequent impact on DOC also shows similar behaviour. Price *et al*.^53^ shows that beam homogeneity significantly varies with curing lamp distance and studies conducted on four commercial LED curing units shows the same trend exhibited by the curing lamp used in this study as shown in Fig. 4. However the clinical scenarios of impact of beam profile is more complex^54^ as it depends on the dentists handling of the curing lamp and further studies might be required to quantitatively estimate the impact.

The polymerisation shrinkage strain response of the embedded FBGs at different location shows the impact of curing lamp beam profile and distance from the specimen. As seen in Fig. 6(a–c) for all the specimens, the FBGs in the central region recorded highest wavelength shifts whereas for the edges the response was slightly lower. For example, the specimen placed at 2 mm from the lamp, the shrinkage strain at the central was 1700 µε where as for the edges it was 1170 µε and 1120 µε respectively, which show a difference of approximately 500 µε between the centre and the edges (for the edges the average of strain on both the edges are taken). Similar trend was evident for other specimen, but the difference in the shrinkage strain between the centre and edges decreases as we increase the distance between the specimen and the lamp. For a distance of 5 mm between the lamp and the specimen the difference in shrinkage strain is approximately 400 µε and while for 10 mm it is 300 µε between the centre region and the edge regions.

The post-gel volumetric shrinkage is calculated from the shrinkage strain assuming uniform shrinkage in all directions (macroscopically isotropic) and is shown in Fig. 7. The FBG array sensor provides an insight to the local post-gel PS distribution across the specimen. However one thing to note here is that the FBG measures post-gel shrinkage, when the composite resin becomes stiffer enough to transfer the shrinkage strain from the composite resin to the embedded FBG. The post-get PS value is only a fraction of the total volumetric shrinkage, which consists of pre-gel and post-gel PS. However post-gel PS is more significant as its results in clinically significant stress on the composite resin and tooth boundaries, where in the pre-gel phase, where most of the shrinkage happens the stress is absorbed by the material itself. For the commercial composite used in this study (Beautifil FO3) the total volumetric shrinkage reported by other researchers is 4.13% which consist of gel and post gel PS. One comparable method for FBG sensing approach is the strain gauge method which also measures post-gel shrinkage^41,42^. The PS studies conducted using strain gauges shows post-gel shrinkage values ranging from 0.66% to 8.87% for different commercial composites resins^42^. However data for post-gel shrinkage of Beautifil FO3 was not available. But from other studies it can be seen than post-gel PS is only a small percentage of total volumetric PS, where this study also confirms.

As seen in Fig. 7, there is a difference of 0.16% in the shrinkage value between the centre and edges (average of right and left edges) for the specimen cured 2 mm away from the lamp, where the difference 0.13% and 0.10% for the specimen cured at 5 mm and 10 mm away from the lamp. This is approximately 30% of the post-gel shrinkage value for all the cases. This is an interesting observation as the difference between the post-gel shrinkage in the centre region and edge region is similar for all the curing conditions, by obtaining the post-gel shrinkage at the centre, the shrinkage at the edge regions can be obtained statistically, provided data for one curing condition is available across all the regions of the specimen. Another point to be noted for the FBG based sensing approach is compared to strain gauge, multiple sensors can be integrated in the same optical fibre facilitating shrinkage measurement at different regions. Though the post-gel PS is different at different regions indicating a strain gradient within the specimen, it hasn’t affected the FBG due to its miniature size. This is reflected in Fig. 8, where the spectral bandwidth of the reflected signals from the FBG sensor before and after curing remains the same indicating no impact of strain gradient.

Given the DOC difference is not significant at different regions of the specimen, the possible reason for the difference in local post-gel PS is due to curing lamp intensity distribution on the curing rate. It can be seen that the curing rate is different for different locations and for different curing distances. It has been previously reported that curing rate is related to shrinkage- higher the curing rate, larger the PS^48^. Our results also show that, there is direct correlation between curing rate and post-gel PS. For example for the case of centre region of specimen cured at 2 mm, the average curing rate (for 20 minutes) is 1.32 times faster than the specimen cured 10 mm away from the lamp. The post-gel PS value at 2 mm distance also reflects the same where it is 1.42 times higher than the 10 mm curing case. Similarly, the difference between average curing rates at the centre of the sample cured at 2 mm is 1.18 times higher than the 5 mm cured one and the corresponding post-gel PS is of 1.17 times higher. The same trend also applies for the edge regions as well the correlation is true across all the specimens. The current study shows that the post-gel PS is inversely proportional to the distance from curing light tip to restoration surface; higher the distance, lower is the shrinkage. Compared to 2 mm distance, there is a reduction of 18.75% and 34.17% shrinkage strain for 5 mm and 10 mm distance cases, similar to Visvanathan^55^, where the polymerisation shrinkage stress at 6 mm distance was 52.94% lower than that of 1 mm distance. Curing rate of photocured dental composites is directly dependent on the power density of the curing lamp^56^; higher the power density, higher is the curing rate. In the current study, the curing rate at 2 mm distance at 100 sec is 25.0% and 33.33% higher than at 5 mm and 10 mm distance.

Thus the results show that the light intensity and intensity distribution plays a crucial role on the polymerisation shrinkage value. Though the DOC change is not that significant with the curing light beam profile, the curing rate and its impact on the post-gel polymerisation shrinkage are significant.

### Potential clinical significance and impact

As the dental curing lamps available in the markets vary in lots of aspects- tip diameter, intensity and irradiance and this will affect the restoration quality. As such the many of the physical characteristics properties, which is a function of the curing lamp characteristics, will differ in laboratory testing conditions and in clinical applications^54,57^. The considerable variation in dental composite formulation, shade filler types and light transmission characteristics, will lead to uncertainties with practitioners on whether their light curing device adequately cure the material they are placing. Also depending on the curing lamp characteristics and cavity boundary conditions, PS stress characteristics will also vary and lead to debonding along the restoration/tooth interface, resulting in marginal gaps and micro-cracking of the restorative material and tooth structure^58^. As demonstrated in the Fig. 7, using the method proposed in this paper, practitioners and researchers can be well informed about the maximum and minimum post-gel PS and the corresponding irradiance and profile of the lamp leading to the composite resin characteristics. Therefore the approach presented in this study can lead to the development of an optimal composite resin-curing lamp characteristics model for quality restorations and might improve their longevity.

Also the excess heat generated by the curing lamp and the exothermic reaction of the composite resin will also have clinical implication as it affects the tooth pulp and soft tissues^59,60^. Different studies have conducted and suggestions are provided to limit the exposure to 20 s to minimise the temperature rise of the pulp^61^. However reducing the exposure time might have impact on the restoration quality. These issues can also be reduced by having a good understanding of the resin PS kinetics and distribution, and the curing lamp irradiance can be optimised to achieve the required polymerisation without having any curing light temperature induced effects.

## Conclusions

FBG sensing based technology is demonstrated as viable technique to measure dental polymerisation kinetics. Post-gel PS of dental composite specimens at multiple locations within specimens was measured and is correlated with local DOC and curing rate. The measured difference in DOC between specimens cured at 2 mm and 10 mm away from the curing lamp was only approximately 2.5%. However, the results show that curing light intensity and intensity distribution, a significant impact on the curing rate and post-gel PS. The measured post-gel PS value was different at different regions of the specimen with highest recorded at the centre compared to the edges. It was evident that there is a clear correlation between curing rate and PS as the local curing rate was proportional to local post-gel PS.
